# Yeast centrosome components form a noncanonical LINC complex at the nuclear envelope insertion site

**DOI:** 10.1083/jcb.201809045

**Published:** 2019-03-12

**Authors:** Jingjing Chen, Jennifer M. Gardner, Zulin Yu, Sarah E. Smith, Sean McKinney, Brian D. Slaughter, Jay R. Unruh, Sue L. Jaspersen

**Affiliations:** 1 Stowers Institute for Medical Research, Kansas City, MO; 2 Department of Molecular and Integrative Physiology, University of Kansas Medical Center, Kansas City, KS

## Abstract

Bipolar spindle formation in yeast requires insertion of centrosomes (known as spindle pole bodies [SPBs]) into fenestrated regions of the nuclear envelope (NE). Using structured illumination microscopy and bimolecular fluorescence complementation, we map protein distribution at SPB fenestrae and interrogate protein–protein interactions with high spatial resolution. We find that the Sad1-UNC-84 (SUN) protein Mps3 forms a ring-like structure around the SPB, similar to toroids seen for components of the SPB insertion network (SPIN). Mps3 and the SPIN component Mps2 (a Klarsicht-ANC-1-Syne-1 domain [KASH]–like protein) form a novel noncanonical linker of nucleoskeleton and cytoskeleton (LINC) complex that is connected in both luminal and extraluminal domains at the site of SPB insertion. The LINC complex also controls the distribution of a soluble SPIN component Bbp1. Taken together, our work shows that Mps3 is a fifth SPIN component and suggests both direct and indirect roles for the LINC complex in NE remodeling.

## Abstract (precis)

How the nuclear envelope is remodeled to facilitate insertion of large protein complexes is poorly understood. Chen et al. use superresolution imaging with bimolecular fluorescence complementation to show that a novel noncanonical linker of nucleoskeleton and cytoskeleton (LINC) complex forms at sites of nuclear envelope fenestration in yeast.

## Introduction

The double lipid bilayer of the nuclear membrane serves as a physical barrier to restrict movement of macromolecules from the cytoplasm to nucleus, or vice versa. Throughout interphase, transport across the nuclear envelope (NE) is facilitated by nuclear pore complexes (NPCs) that are located at sites where the inner and outer nuclear membranes (INM and ONMs) are contiguous, known as the pore membrane. In fungi, as well as in rapidly dividing cells such as *Drosophila melanogaster* and *Caenorhabditis elegans* embryos, the INM and ONM also come together to form a fenestra at or near the microtubule-organizing center (Funakoshi et al., 2011; Smoyer and Jaspersen, 2014), which is known as the centrosome in metazoans and the spindle pole body (SPB) in yeast. Unlike most metazoans, the NE does not break down in these systems during mitosis, so integration of the SPB into the NE in yeast, for example, is necessary so that microtubules can form a mitotic spindle to segregate the genome within the nucleus while simultaneously nucleating cytoplasmic microtubules that orient the nucleus for delivery of a genome into each of the daughter cells.

**Figure 1.**
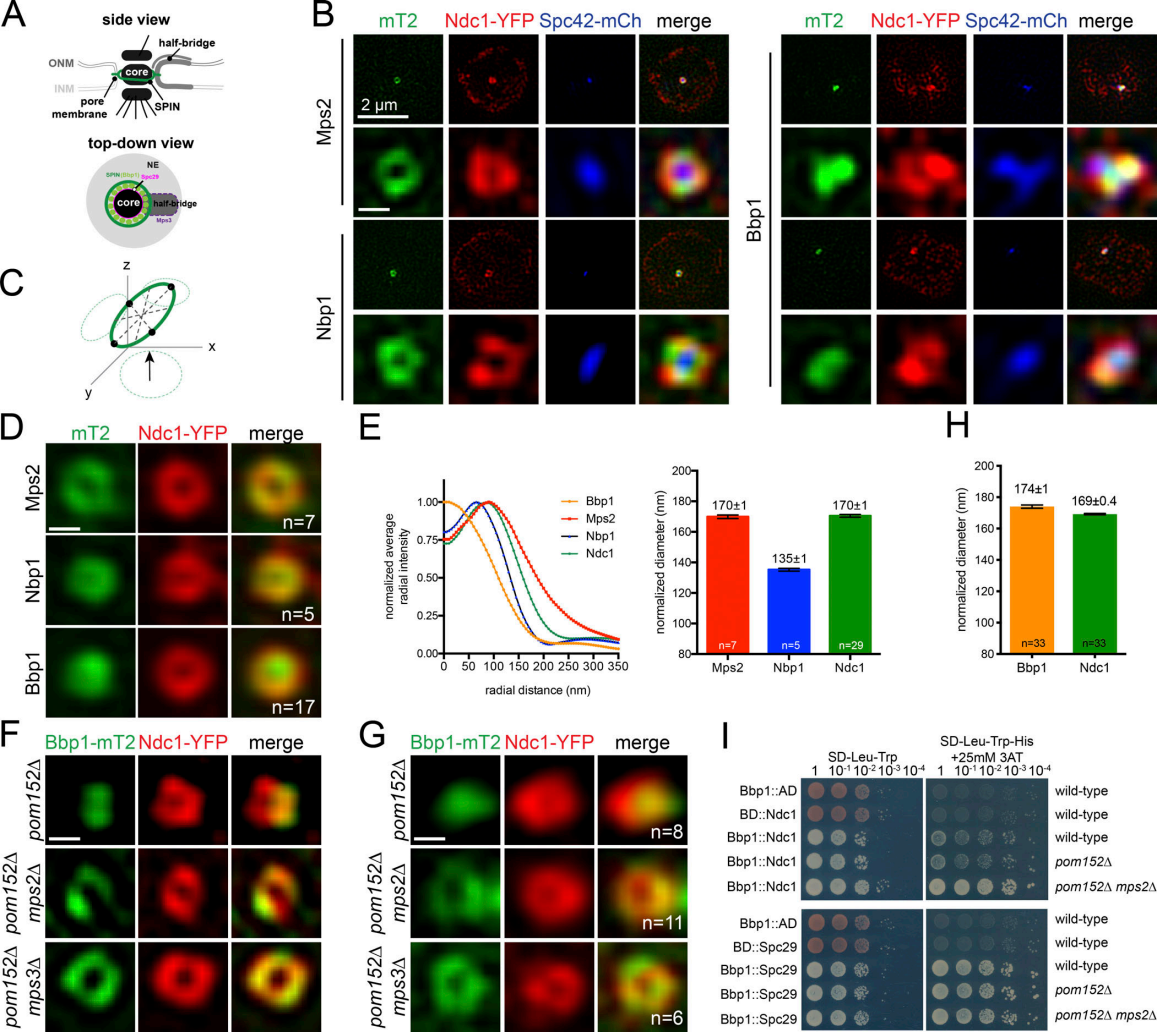
**Radial distribution of SPIN components at the SPB. (A)** SPB schematic showing the core, SPIN, and half-bridge based on a side-on or top-down view of the SPB. Through Bbp1 binding to Spc29, the SPIN is thought to form a pore for SPB insertion and anchorage. **(B)** Representative SIM images of nuclei (bar, 2 µm) with Ndc1-YFP (red) and Spc42-mCherry (blue) to detect the SPB ring and core, respectively, along with the indicated protein tagged with mT2 (green; SLJ11171, Mps2; SLJ10898, Nbp1; SLJ10635, Bbp1). Ndc1-YFP is also present at NPCs (Chial et al., 1998). **(C and D)** As shown in C, averaged images (D) were generated by realigning multiple SPB rings as indicated (n) in 3D (see Fig. S1, A and B). **(E)** Fluorescence profiles of SPIN components from the center of the SPB outwards, based on the projections in D. Average ring diameter was determined in aligned images based on the center of Gaussian fits of fluorescence intensity. Because Ndc1-YFP diameter varied by ≤20 nm between different strain isolates, values were normalized using Ndc1-YFP values. Errors were determined by Monte Carlo analysis. **(F)** Localization of Ndc1-YFP (red) and Bbp1-mT2 (green) in asynchronously grown *pom152Δ* (SLJ12302), *pom152Δ mps2Δ* (SLJ10998), and *pom152Δ mps3Δ* (SLJ10534) strains by SIM. **(G and H)** Averaged images were generated as in D, and average ring diameter was determined from the mutants, as in E. **(I)** Pairwise protein interactions between Bbp1 fused to the Gal4-binding domain (BD) and Ndc1 or Spc29 fused to the Gal4 activation domain (AD) expressed from centromeric plasmids were tested by serial dilution assays in the yeast two-hybrid system in wild-type (SLJ1644), *pom152Δ* (SLJ12623), and *pom152Δ mps2Δ* (SLJ12624). Empty binding domain and activation domain vectors were used as controls. Growth on media lacking tryptophan (Trp), leucine (Leu), and histidine (His) that also contained 25 mM 3AT (right) indicates an interaction, while growth on Trp-Leu is a plating control. Bars, 100 nm.

In budding yeast, the SPB is anchored in a fenestrated region of the NE throughout the cell cycle (Rüthnick and Schiebel, 2016; Cavanaugh and Jaspersen, 2017). Genetic analysis suggests that SPB incorporation into the NE requires at least four factors: a soluble SPB protein, Bbp1; an amphipathic domain-containing protein, Nbp1; the dual SPB-NPC transmembrane protein Ndc1; and a Klarsicht-ANC-1-Snye-1 homology (KASH)–like protein, Mps2 (Winey et al., 1991; Chial et al., 1998; Muñoz-Centeno et al., 1999; Schramm et al., 2000; Araki et al., 2006). Known as the SPB insertion network (SPIN), these components display extensive genetic and physical interactions and are thought to form a donut-like structure around the core SPB that anchors it in the NE (Fig. 1 A; Rüthnick et al., 2017). Loss of SPIN function through conditional mutations results in an inability to insert the newly duplicated SPB into the NE, indicating an additional role in NE fenestration (Winey et al., 1991; Chial et al., 1998; Muñoz-Centeno et al., 1999; Schramm et al., 2000; Araki et al., 2006). Interestingly, specific NPC components genetically interact with the SPIN, leading to the idea that NPCs and SPBs share common regulators or insertion factors, including NPC components themselves (Chial et al., 1998; Lau et al., 2004; Sezen et al., 2009; Witkin et al., 2010; Casey et al., 2012; Chen et al., 2014; Rüthnick et al., 2017). How SPIN components or NPCs lead to NE fenestration is not understood. In both mammals and yeast, data suggest that the conserved family of Sad1-UNC-84 (SUN) domain proteins also play a role in NE insertion of SPBs or NPCs (Friederichs et al., 2011; Talamas and Hetzer, 2011; Fernández-Álvarez et al., 2016; Bestul et al., 2017), suggesting that SPIN components might interact with other NE factors to remodel the membrane.

Central to understanding how complexes such as the SPB and NPC are assembled and anchored in the membrane is the need to develop rigorous, reproducible methods to compare NE-associated protein structures at high resolution. Here, we describe how structured illumination microscopy (SIM), iterative 3D single-particle analysis (SPA), and bimolecular fluorescence complementation (BiFC) can be combined to study the organization of SPIN proteins at the SPB in wild-type and mutant cells. This approach led to the surprising discovery that the SPIN forms at least two domains, one that contains Bbp1 and a Bbp1-independent region. We show this is due, at least in part, to the budding yeast SUN protein Mps3, which forms an atypical SUN-KASH–like complex with Mps2.

## Results and discussion

### Radial distribution of SPIN components using 3D particle averaging

The SPB is a multilayered cylindrical organelle permanently embedded in the NE. A modified region of the NE, known as the half-bridge, is associated with one side of the SPB (Fig. 1 A). The SPIN component Ndc1 can be seen surrounding the SPB core (Spc42) using SIM in diploid cells, which have a diameter (160 nm) that is above the resolution limit (∼100 nm; Fig. 1, A and B). We refer to the donut-like localization pattern as a ring or toroid, although it is important to note that at high resolution, the toroid is not homogeneous (Fig. S1, A and B).

To understand the role of the SPIN in NE fenestration at the SPB, we tagged SPIN components at their endogenous loci with mTurquoise2 (mT2), taking care to ensure that tagging did not significantly affect yeast growth or ploidy, and used SIM in asynchronously growing cells to examine localization relative to the SPB toroid (Ndc1-YFP) and core (Spc42-mCherry; Fig. 1 B). We computationally aligned, reconstructed, and normalized images of randomly oriented SPBs using Ndc1-YFP to facilitate comparison between samples (Fig. 1 C and Fig. S1, A and B). If any heterogeneity observed by SIM in individual images occurred at a reproducible location, such as proximal or adjacent to the half-bridge, it would be possible to align gaps to a known SPB reference (i.e., Mps3, Sfi1) using this approach. The simplest explanation as to why gaps in intensity do not align is that they are caused by noise in imaging at this resolution (Fig. S1 B), similar to other ring-like structures (e.g., Mennella et al., 2012; Szymborska et al., 2013; Gartenmann et al., 2017).

Mps2-mT2 and Nbp1-mT2 largely colocalized with Ndc1-YFP at the SPB toroid in both individual images (Fig. 1 B) and averaged toroids (Fig. 1 D). The sizes of Ndc1-YFP and Mps2-mT2 toroids are identical to each other (170 ± 1 nm) and to estimates of SPB diameter (160 nm in diploids) determined by EM (Byers and Goetsch, 1974; Li et al., 2006), validating our realignment and normalization protocol. In contrast, the Nbp1 toroid diameter (135 ± 1 nm) was 20% smaller (Fig. 1 E), perhaps explaining the difficulty in visualizing Nbp1 as a toroid using the longer-wavelength YFP fluorophore (Burns et al., 2015).

Unlike other SPIN components, Bbp1-mT2 typically did not localize to a toroid but rather formed one or two large puncta (Fig. 1 B). In rare instances (7 of 63), a ring-like distribution of Bbp1-mT2 was detected in unbudded G1, medium-budded S phase, and large-budded mitotic cells, making it unlikely that Bbp1-mT2 toroidal distribution is cell cycle regulated. Therefore, we considered the possibility that the distribution of Bbp1-mT2 was spatially controlled by its primary binding partner, Mps2 (Schramm et al., 2000; Kupke et al., 2017). *MPS2* can be deleted in yeast strains lacking the transmembrane nucleoporins *POM34* or *POM152* (Witkin et al., 2010; Kupke et al., 2011; Katta et al., 2015). In the absence of *MPS2*, Bbp1-mT2 distributed to ring-like structures that colocalize with Ndc1-YFP (Fig. 1, F and G; and Fig. S2, A and B). The size of the Bbp1-mT2 toroid (174 ± 1 nm) in the double mutants was similar to that of Ndc1 and Mps2 (Fig. 1 H), suggesting that loss of *MPS2* might allow Bbp1 to bind to another SPIN or SPB component at the membrane region surrounding the SPB core.

We examined binding between Bbp1 and Ndc1 or Spc29 in the yeast two-hybrid system in wild-type cells or in cells lacking *MPS2* as a test of this idea. Binding efficiency was assayed using the *HIS3* reporter in a serial dilution assay (Uetz et al., 2000). Bbp1 binding to Ndc1, but not to Spc29, increased in cells lacking *MPS2* (Fig. 1 I). This specific effect of Mps2 on the Bbp1–Ndc1 interaction, together with our localization data, supports the idea that the SPIN toroid has at least two domains: a region that includes Mps2, Nbp1, and Ndc1 and a second that also contains Bbp1.

### Mps3 is a component of the SPB toroid and bridge

Mps3 is a highly divergent SUN domain–containing protein that binds to the C terminus of Mps2 in vitro and in vivo (Jaspersen et al., 2006). Although Mps2 lacks a canonical KASH motif (broadly defined as a short C-terminal tail that terminates in PX), the organization of Mps3 at the INM and Mps2 at the ONM of vegetative cells is highly similar to that of SUN-KASH proteins (see Fig. 3, A–C and G; Muñoz-Centeno et al., 1999; Jaspersen et al., 2002; Nishikawa et al., 2003; Smoyer et al., 2016), suggesting that Mps2 and Mps3 form a linker of nucleoskeleton and cytoskeleton (LINC) bridge across the INM and ONM. Conditional mutant alleles (Fig. S2 C) in the Mps3 SUN domain, the Mps2 C terminus (*mps2-381*), or a dominant allele of *MPS3* give rise to similar errors in SPB assembly, including initiation of SPB formation (Jaspersen et al., 2006), NE anchorage of the SPB (Fig. S2 D), and SPB insertion (Fig. S2, E and F; Friederichs et al., 2011). Deletion of *MPS3*, like *MPS2*, also results in redistribution of Bbp1-mT2 to a toroid (Fig. 1, F and G; and Fig. S2, A and B), suggesting that Mps3, including its luminal domain, at least indirectly affects SPIN organization and SPB fenestration.

**Figure 2.**
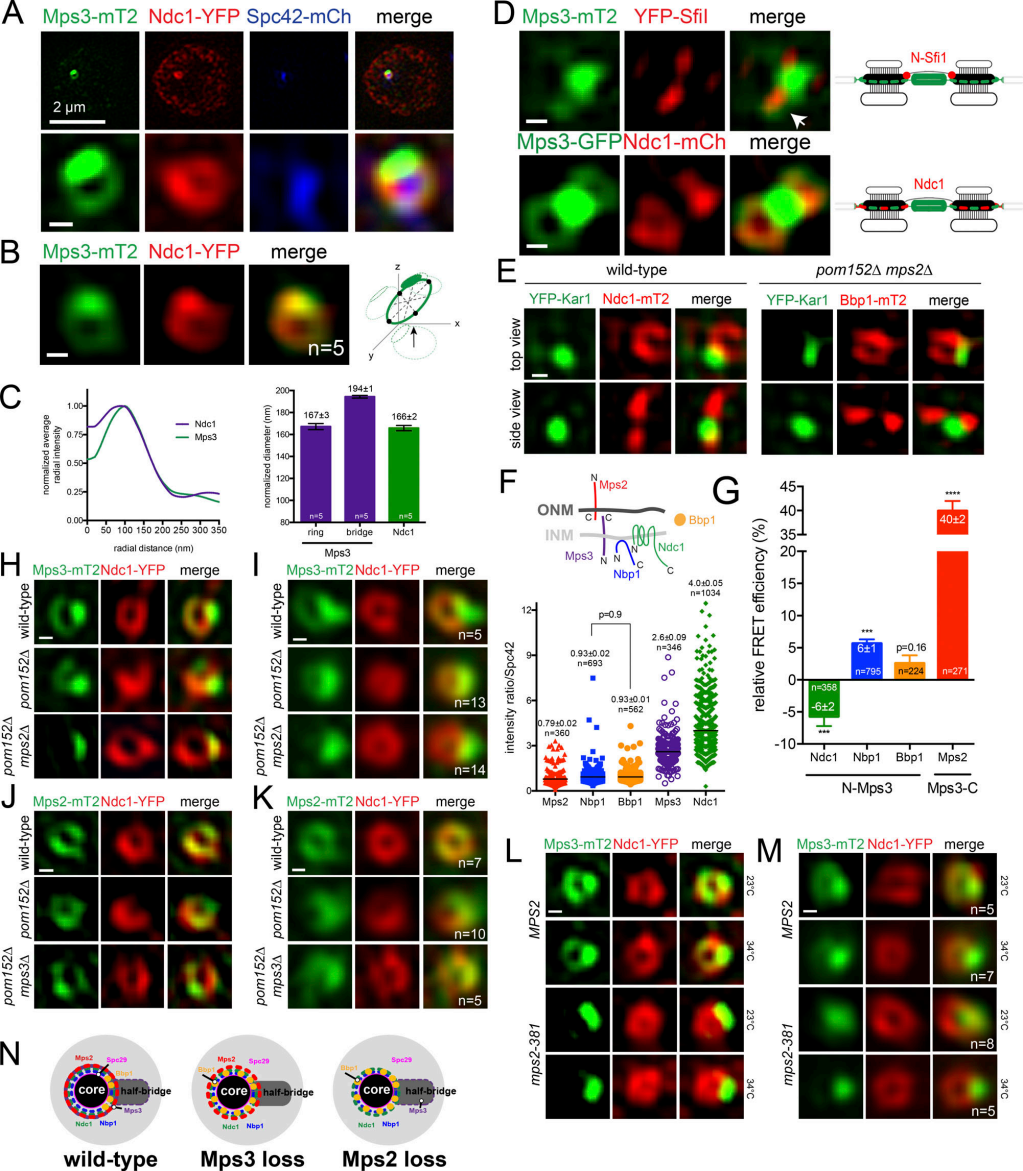
**Mps3 localization to the SPB toroid is Mps2 dependent. (A and B)** Representative SIM image of nucleus from a cell containing Ndc1-YFP (red), Spc42-mCherry (blue), and Mps3-mT2 (green; SLJ10636). Bar, 2 µm. The SPB region (A) and averaged ring from the indicated number (n) of images (B) are below. **(C)** Ring diameter, as in Fig. 1 E. Because Mps3-mT2 was anisotropic, its diameter varied based on the region selected for analysis; shown are the ring region only and ring and the half-bridge domain. Errors were determined by Monte Carlo analysis. **(D)** In G1 cells (SLJ12060), Mps3-mT2 (green) is present between the YFP-Sfi1 (red) foci that mark the ends of the extended bridge and in a ring (arrow; also in Fig. S3 A). Mps3-GFP (green) colocalizes with Ndc1-mCherry (red) at toroids (SLJ5496). Schematics illustrate protein distribution at SPBs. **(E)** Top-down and side-on view from wild-type (SLJ10001) and *pom152Δ mps2Δ* (SLJ12620) strains by SIM showing YFP-Kar1 (green). **(F)** Topology of Ndc1, Mps2, and Mps3. Nbp1 interacts with the nuclear side of the membrane via its amphipathic helix (Kupke et al., 2011). Bbp1 is soluble (Schramm et al., 2000). Because acceptor photobleaching FRET is sensitive to protein abundance, we determined levels of Mps2-mT2 (SLJ8065), Nbp1-mT2 (SLJ12020), Bbp1-mT2 (SLJ11903), Mps3-mT2 (SLJ8835), and Ndc1-mT2 (SLJ7941) in asynchronously growing haploid cells at the SPB relative to the amount of Spc42-YFP. Long bars depict average values, which are listed with SEM based on the number of points shown. P values were calculated using two-sided Student’s *t* test; all are significant (P < 0.0001) except for Nbp1-mT2 and Bbp1-mT2. **(G)** Binding between Mps3 and SPIN components was analyzed at the SPB using acceptor photobleaching FRET in asynchronously growing cells. Average FRET efficiency in the number of cells analyzed is listed along with SEM. Negative FRET values are most likely due to bleaching of the donor, since we excluded cells in which the SPBs moved. P values were determined using the two-sided Student’s *t* test compared with the donor-only control. ***, P = 0.0005; ****, P < 0.0001. **(H–K)** Individual SIM (H and J) and averaged (I and K) images showing localization of Ndc1-YFP (red) and the distribution of Mps3-mT2 (H and I) or Mps2-mT2 (J and K; green) in wild-type (SLJ10636; SLJ11171), *pom152Δ* (SLJ11071; SLJ12370), and *pom152Δ mps2Δ*/*pom152Δ mps3Δ* (SLJ10535; SLJ11173). **(L and M)** Distribution of Mps3-mT2 (green) along with Ndc1-YFP (red) in individual (L) or averaged (M) SIM images of wild-type (SLJ12772) and *mps2-3*81 (SLJ12616) cells grown at 23°C or shifted to 34°C for 3 h. **(N)** Top-down SPB view summarizing Mps3 and SPIN localization in wild-type cells and in mutants lacking *MPS2* or *MPS3*. Bbp1 localizes near the bridge in wild-type cells (Burns et al., 2015), but the Mps3 bridge tether is unknown. While Kar1 has been proposed to be a KASH-like protein, it contains a single amino (F) acid in the luminal space. Bars, 100 nm unless indicated otherwise.

To test if Mps3 shows a toroidal distribution like Mps2, we examined Mps3-mT2 localization by SIM in a diploid strain containing Ndc1-YFP and Spc42-mCherry. In individual and merged images (Fig. 2, A and B), Mps3-mT2 surrounded the SPB similar to the ring-like distribution recently described for the fission yeast SUN protein Sad1 (Bestul et al., 2017). Why the toroidal distribution of Mps3 was not observed by Rüthnick et al. (2017) is unknown, but it may relate to the use of overexpressed *SPC42* and *SPC29*, which alter SPB architecture (Donaldson and Kilmartin, 1996; Schramm et al., 2000). Mps3’s distribution was different from that of other SPIN components in that a significant fraction of Mps3-mT2 appeared as a large focus on one side of the SPB or between duplicated SPBs; this corresponded to the bridge, as shown by colocalization with the cytoplasmic bridge protein YFP-Sfi1 (Fig. 2 D). Alignment and normalization of toroids revealed that Mps3 diameter in the x direction that does not include the bridge is 167 ± 3 nm, while the length in the y axis is 194 ± 1 nm (Fig. 2 C).

Toroid formation is not a general feature of bridge components, as we did not detect other soluble or membrane proteins such as Sfi1 or Kar1 surrounding the SPB core (Fig. 2, D and E; and Fig. S3 A). Thus, Mps3 uniquely exists in three populations: the INM (Fig. S3 A), the SPB bridge, and the toroid surrounding the core SPB. Quantitation of the average realigned fluorescence distribution indicates that roughly half (45 ± 5%, *n* = 5) of Mps3 protein at the SPB is located in the toroid, with the remainder (55 ± 5%, *n* = 5) densely packed in the bridge region extending away from the core SPB. Based on its distribution at the toroid, effect on Bbp1 localization, and role in SPB insertion and Mps2 binding, we propose that Mps3 is a novel component of the SPIN.

### Mps3 toroid formation is Mps2 dependent

Using fluorescence resonance energy transfer (FRET), we assayed Mps3’s interactions with other SPIN components, taking into account the relative abundance of donor and acceptor proteins and protein topology, which both affect FRET (Fig. 2 F). We did not detect FRET between YFP-Mps3 and Ndc1-mT2, and our FRET between YFP-Mps3 and Bbp1-mT2 was not statistically significant compared with controls (Figs. 2 G and S2 G). However, FRET between YFP-Mps3 and Nbp1-mT2 was 5.7 ± 0.7% (*n* = 795), similar to FRET levels observed between other SPIN components (Figs. 2 G and S2 G). We were unable to test FRET between the N termini of Mps2 and Mps3 because the tagged strains were lethal in combination. The 39.9 ± 2.1% (*n* = 271) FRET between the C termini of Mps3 and Mps2 was more than double that of any other protein pair examined, including our positive FRET control (Figs. 2 G and S2 G), consistent with the idea that Mps2 and Mps3 form a LINC-like complex at the SPB. Moreover, the very high FRET strongly suggests that a single Mps3 C terminus (donor) interacts with multiple copies of the Mps2 C terminus (acceptor), an alternative high-stoichiometry Mps2–Mps3 complex compared with the SUN2-KASH1/2 trimer (Sosa et al., 2012). While this is perhaps not surprising given that Mps3 lacks key residues that mediate the traditional SUN–KASH interface (Sosa et al., 2012), it raises the possibility that SUN proteins, particularly those involved in centrosome tethering or those from organisms such as yeast that have lost intermediate filaments during evolution, may interact with KASH-like proteins using alternative mechanisms (Meier, 2016).

How does the luminal Mps2–Mps3 interaction affect their distribution at the SPB? Examination Mps2-mT2 or Mps3-mT2 in cells lacking *MPS3* or *MPS2*, respectively, showed that Mps3 was lost specifically from the toroid in *pom152Δ mps2Δ* or *pom34Δ mps2Δ* cells (Fig. 2, H–K; and Fig. S3, B–E). This loss is not due to *pom152Δ* or *pom34Δ*, nor is it caused by gross structural abnormalities at the SPB, as other SPIN components such as Ndc1 and Npb1 localized to the toroid in mutant cells (Fig. S3, F and G). The finding that SUN protein (Mps3) localization is dependent on the KASH-like protein (Mps2) but Mps2 localization is Mps3 independent is distinct from canonical LINC interactions in which the SUN protein is required for KASH protein recruitment. Nonetheless, the luminal Mps2–Mps3 interaction is required to recruit Mps3 to the toroid, as truncation of Mps2 in the luminal region (*mps2-381*) results in loss of Mps3-mT2 from the toroid but not the bridge (Fig. 2, L and M; and Fig. S2 C), providing evidence that Mps2–Mps3 form a LINC complex.

### Mps2 binding to Mps3 and Bbp1 in the toroid

Two forms of Mps3 exist at the SPB: a toroid-specific population of Mps3 that requires Mps2 for its formation and/or stabilization, and a second population that localizes to the bridge independently of interaction with Mps2 (Fig. 2 N). The Mps2 binding protein Bbp1 also shows an unexpected restricted distribution within the toroid (Fig. 2 N). That Bbp1 is able to localize to the toroid in cells lacking *MPS3* suggests that Mps3 and Bbp1 organization are coordinated, possibly through Mps2 binding, which also causes Bbp1 reorganization when removed (Fig. 2 N).

**Figure 3.**
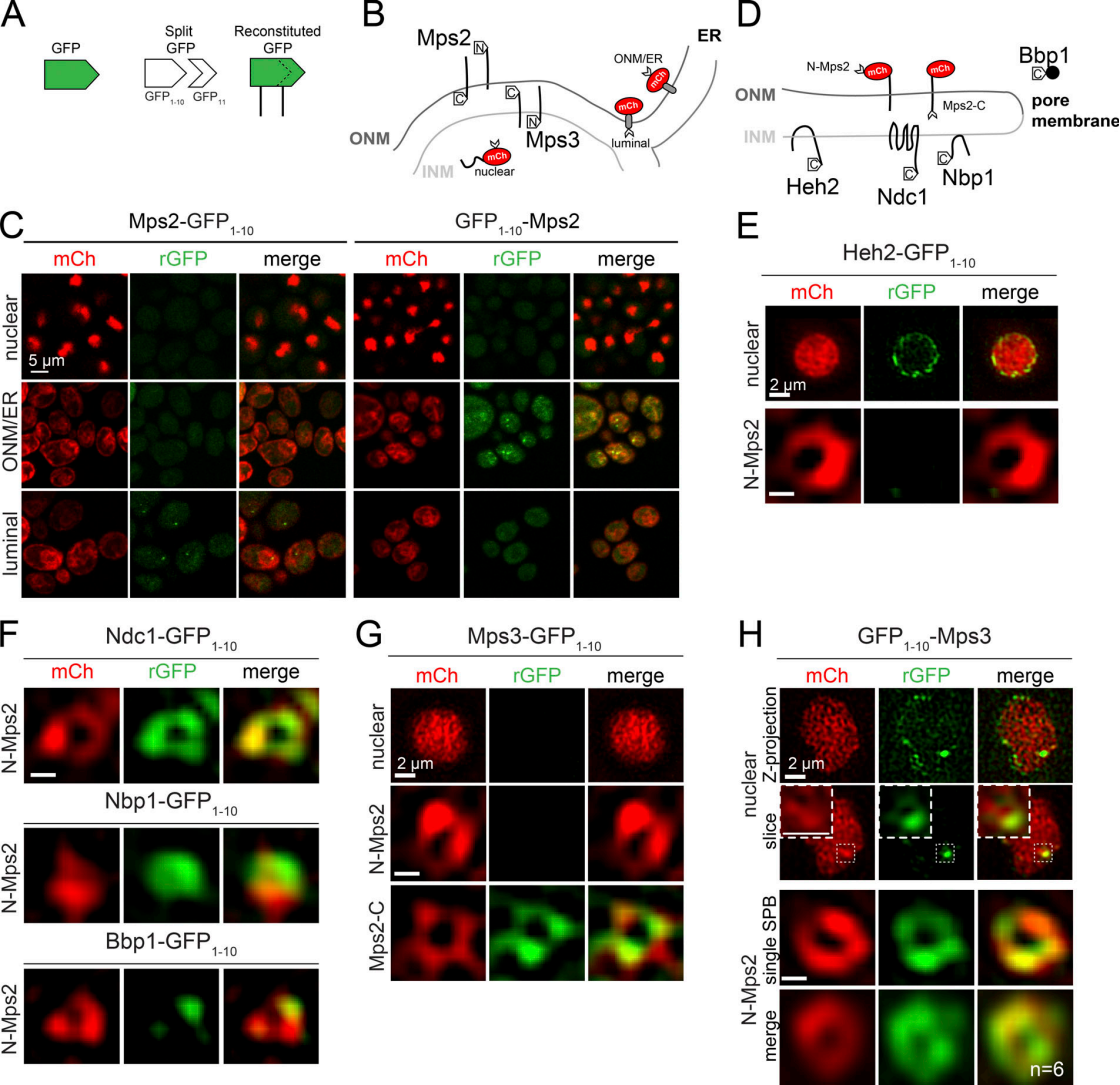
**High-resolution mapping of Mps2 binding domains. (A)** GFP can be split into two nonfluorescent halves, GFP_1–10_ and GFP_11_, which can reconstitute fluorescence if present in the same subcellular compartment (Cabantous and Waldo, 2006). **(B)** Schematic illustrating reporters to detect nuclear (GFP_11_-mCherry-Pus1), ONM/ER (GFP_11_-mCherry-Scs2TM), and luminal (mCherry-Scs2TM-GFP_11_) distribution. The distribution/topology of Mps3 using this system has been previously reported (Smoyer et al., 2016). **(C)** Topology of Mps2 in the NE was tested using the split-GFP system. Fluorescence of the reporter (red) and from rGFP (green) are shown. **(D)** Schematic illustrating N-terminal (GFP_11_-mCherry-Mps2) and C-terminal (mCherry-Mps2-GFP_11_) reporters to test Mps2 binding interactions at the NE and pore membrane. **(E–H)** SIM from cells expressing the nuclear, N-terminal, and/or C-terminal Mps2 reporters (red) with Heh2-GFP_1–10_ (SLJ8138/12773; E), Ndc1-GFP_1–10_ (SLJ12825), Nbp1-GFP_1–10_ (SLJ12826), Bbp1-GFP_1–10_ (SLJ12866; F), Mps3-GFP_1–10_ (SLJ9399/12476/12990; G), or GFP_1–10_-Mps3 (SLJ8577/12474; H), as indicated. Although GFP_11_-mCh-Pus1 is present through the nucleoplasm and rGFP can be seen at the INM with Heh2-GFP_1–10_ and GFP_1–10_-Mps3, only GFP_1–10_-Mps3 results in rGFP signal at the SPB, illustrated in magnified images of a single nuclear slice in H. Individual and averaged images of rGFP rings in GFP_1–10_-Mps3/GFP_11_-mCherry-Mps2 are also shown in H. Bars, 100 nm unless indicated otherwise.

We used a previously described BiFC assay to examine Mps3, Bbp1, and Mps2 distribution and topology in the NE (Smoyer et al., 2016). In our split-GFP assay, reconstituted GFP (rGFP) can be detected if proteins fused to GFP_1–10_ and GFP_11_ are in the same cellular compartment (Fig. 3 A). Based on the location of the GFP_1–10_ tag, Mps3 can associate with either the nuclear (GFP_11_-mCherry-Pus1) or luminal (mCherry-Scs2TM-GFP_11_) reporter (Fig. 3, G and H; Smoyer et al., 2016), consistent with the expected distribution and topology of a SUN protein. Analysis of Mps2 confirmed a KASH-like distribution: the protein is confined to the ONM with its N terminus facing the cytoplasm and C terminus in the luminal space (Fig. 3 C).

By combining split-GFP with SIM, we next dissected the spatial localization of discrete protein–protein interactions with Mps2. To show specificity, we verified that signal from known INM proteins such as Heh2-GFP_1–10_ was detected with the nuclear reporter (GFP_11_-mCherry-Pus1) at the INM but not with the N-terminal Mps2 reporter (GFP_11_-mCherry-Mps2) at the SPB (Fig. 3, D and E). However, GFP_11_-mCherry-Mps2 combined with Ndc1-GFP_1–10_ and Nbp1-GFP_1–10_ resulted in a rGFP ring largely, but not completely, overlapping with mCherry at the SPB toroid (Fig. 3 F). Importantly, Bbp1-GFP_1–10_, did not interact with GFP_11_-mCherry-Mps2 throughout the toroid but formed a specific rGFP focus (Fig. 3 F). Using a C-terminal Mps2 reporter (mCherry-Mps2-GFP_11_) with Mps3-GFP_1–10_, we observed that the luminal SUN-KASH–like interaction occurs around the SPB toroid (Fig. 3 G). These data support the idea that Mps2 interactions are spatially distinct, with Mps2–Mps3 complexes around the toroid and more restricted Mps2–Bbp1 complexes.

### Role of the Mps3 N terminus in SPB insertion

Based on the canonical organization of SUN and KASH proteins in the INM and ONM, respectively, we anticipated that BiFC between Mps2 and Mps3 N termini would be negative. However, GFP_11_-mCherry-Mps2 together with GFP_1–10_-Mps3 resulted in a rGFP toroid similar to mCherry-Mps2-GFP_11_ and Mps3-GFP_1–10_ (Fig. 3, G and H). One model to explain this result is that the LINC-like complex might fold back on itself in a hairpin specifically at the SPB pore membrane such that the N and C termini of Mps2–Mps3 both interact or are within close proximity.

**Figure 4.**
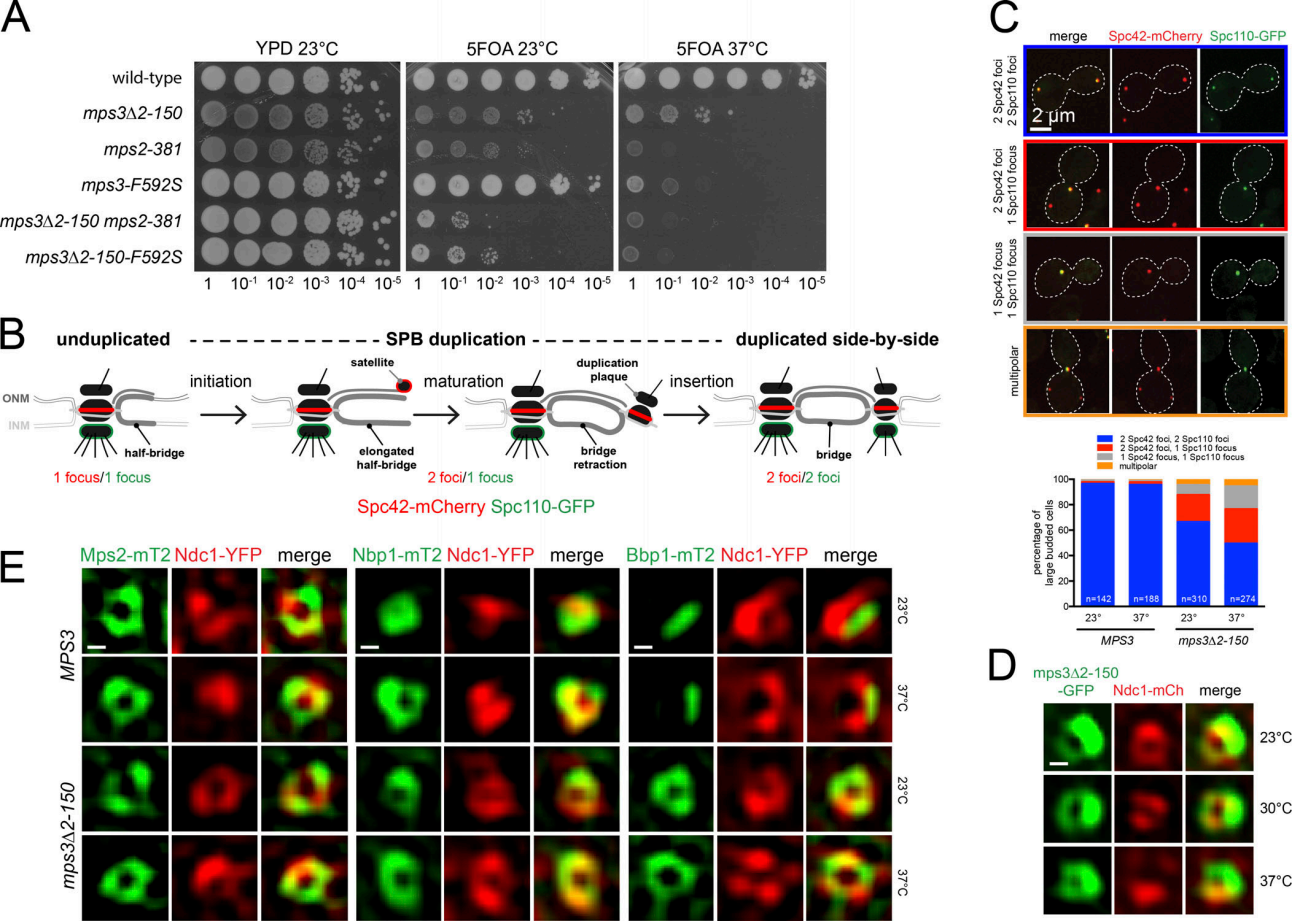
**Role of the Mps3 N terminus in SPB duplication. (A)** Serial dilution assay to test growth of wild-type and single and double mutants containing *pURA3-MPS3* (SLJ1053) on 5-fluoro-orotic acid at 23°C (3 d) and 37°C (2 d). As a control, cells were also stamped to YPD at 23°C (2 d). Note, *mps3Δ2-150*, *mps3-F592S*, and *mps2-381* all encode stable proteins (Jaspersen et al., 2006; Gardner et al., 2011). **(B)** Schematic of SPB duplication pathway from unduplicated to duplicated side-by-side SPBs. Incorporation of Spc110 (green) into the new SPB requires its insertion into the NE, whereas Spc42 (red) localizes to the new SPB early in duplication before insertion. Defects in different steps of SPB duplication can be determined by counting the number of Spc42 and Spc110 foci (Pereira et al., 1999). **(C and D)** Images showing distinct localization patterns of Spc42-mCherry (red) and Spc110-GFP (green). The percentage of *MPS3* (SLJ12982) or *mps3Δ2-150* (SLJ12981) cells grown at 23°C or shifted to 37°C for 3 h with each is shown below. (D) SIM of mps3Δ2-150-GFP (green) and Ndc1-mCherry (red) from cells (SLJ12541) grown at 23°C or shifted to 30° or 37°C for 3 h. **(E)** Localization of Mps2-mT2 (SLJ10472/SLJ13023), Nbp1-mT2 (SLJ8341/SLJ13024), and Bbp2-mT2 (SLJ9231/SLJ13025; green) to SPB toroid by SIM using Ndc1-YFP (red) as a reference, in *MPS3* or *mps3Δ2-150* grown at 23°C or shifted to 37°C for 3 h. Bars, 100 nm unless indicated otherwise.

If Mps2 and Mps3 form a hairpin though their N termini, then failure of hairpin formation through N-terminal mutations in either protein should result in SPB insertion defects. Most *mps2* alleles defective in SPB duplication are located in the N terminus; however, a role for the Mps3 N terminus has not been tested. As shown in Fig. 4 A, deletion of the Mps3 N terminus (*mps3Δ2-150*) results in slow growth compared with wild type. That it exacerbates the growth defect of *mps2-381* or *mps3-F592S* (a mutation in a conserved SUN domain residue; Fig. S2 C) suggests that the N and C termini of Mps3 act cooperatively in SPB function. To further test for SPB insertion defects in *mps3Δ2-150* mutants, we took advantage of the fact that Spc110 incorporation into the new SPB requires its NE insertion (Fig. 4 B). Cells defective in SPB insertion, such as *MPS2* mutants, arrest with large budded cells containing two foci of Spc42-mCherry but only a single focus of Spc110-GFP (Schramm et al., 2000; Sezen et al., 2009). Analysis of *mps3Δ2-150* mutants using this assay confirmed a role for the N terminus in SPB insertion (Fig. 4 C).

At a molecular level, mps3Δ2-150-GFP localizes to the toroid and to the bridge (Fig. 4 D), indicating that the luminal SUN-KASH–like interaction is not only necessary (Fig. 2, L and M) but is also sufficient to mediate Mps3’s ring distribution. Interestingly, we find that Mps2-mT2, Nbp1-mT2, and Ndc1-YFP localization are unaffected in *mps3Δ2-150* but that Bbp1-mT2 redistributes to the toroid (Fig. 4 E). This supports the idea that the Mps3 N terminus restricts Bbp1 from the toroid, providing evidence that both luminal and extraluminal interactions between Mps2 and Mps3 influence SPIN organization.

### Mps2–Mps3 binding during SPB duplication

**Figure 5.**
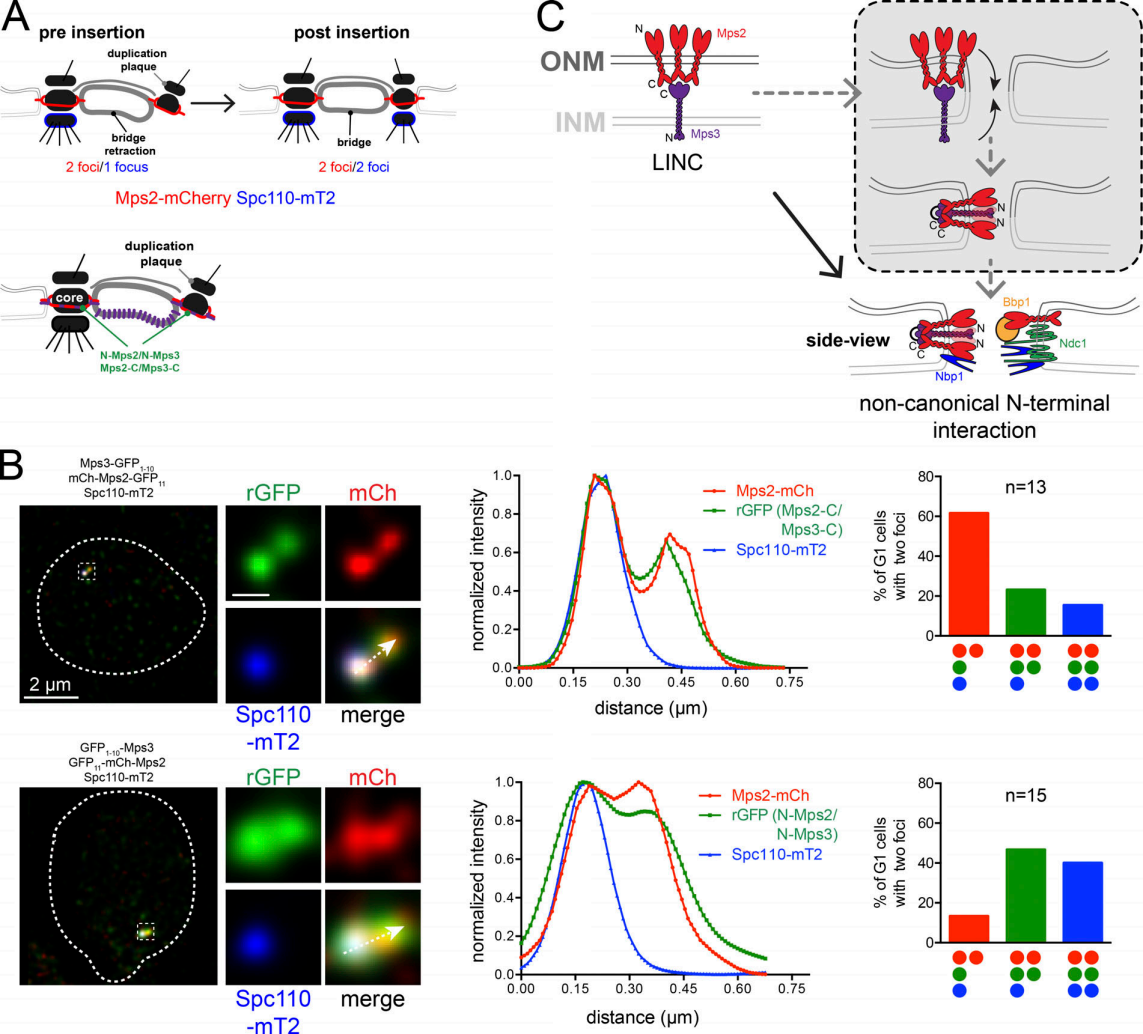
**Interaction of Mps2–Mps3 N and C termini during SPB duplication. (A)** Schematic of part of SPB duplication pathway as in Fig. 4 B. Spc110 (blue) and Mps2 (red) are shown; the localization of Mps3 (purple) is also shown along with the locations where rGFP signal between N-Mps2/N-Mps3 and Mps2-C/Mps3-C pairs (green) was observed. **(B)** Three-color imaging of asynchronously growing cells (SLJ12884/SLJ13021) expressing N- or C-terminal Mps2 split-GFP reporters (red) in strains with GFP_1–10_-Mps3 or Mps3-GFP_1–10_ to assay binding by rGFP (green) relative to SPB insertion, determined by Spc110-mT2 incorporation (blue). A linescan across the magnified SPB region from a G1 cell undergoing SPB duplication illustrates that the rGFP is detected at the distal SPB before Spc110. G1 cells containing two Mps2 foci were quantitated and plotted based on the percentage that also contained one or two foci of rGFP and Spc110. Note that all observed combinations of SPBs with two foci are shown along with the number of G1 cells (Mps2-C/Mps3-C; N-Mps2/N-Mps3). Bar, 2 µm (left); 100 nm (center). **(C)** Mps2–Mps3 localize to the ONM and INM, forming a LINC through their luminal domains, which interact in vitro (Jaspersen et al., 2006) and in vivo (Fig. 2 G), although our FRET data suggest an alternative high-stoichiometry complex compared with typical SUN-KASH trimers. Linkage of Mps2–Mps3 N termini to form a hairpin is noncanonical. We speculate that formation of this hairpin facilitates NE fenestration and/or stabilizes the NE pore at the SPB together with other SPIN components shown (gray box).

An attractive aspect of Mps2–Mps3 hairpin formation is its possible role in membrane remodeling or stabilization at the new SPB before, or during, NE insertion. To determine when during SPB duplication Mps2 and Mps3 N and C termini interact, we analyzed binding based on rGFP in strains containing Spc110-mT2. The presence of one spot of Spc110 suggests that insertion of the new SPB has not yet occurred, while two spots of Spc110 represent postinsertion steps. This marker was combined with various combinations of split-GFP constructs to determine at high spatial resolution whether interactions occur before or after insertion (Fig. 5 A). With both GFP_11_-mCherry-Mps2/GFP_1–10_-Mps3 and mCherry-Mps2-GFP_11_/Mps3-GFP_1–10_, we obtained similar results: we never observed two foci of Spc110 before rGFP was observed at the new SPB (Fig. 5 B). Thus, Mps2 and Mps3 N and C termini interact at the new SPB before Spc110 is assembled, providing evidence that a novel hairpin structure between the Mps2–Mps3 LINC facilitates NE insertion at the SPB (Fig. 5 C).

Our results show that Mps2–Mps3 binding does not tether the bridge to the SPB (Jaspersen et al., 2006), but instead points to a novel role in INM-ONM fusion and/or stabilization. Our data here and in previous work (Jaspersen et al., 2006) demonstrate clear evidence for luminal linkage between Mps2 and Mps3. The notion of an extraluminal interaction between N termini was unexpected, and it raises the interesting possibility that other SUN-KASH/KASH–like complexes form similar hairpins. In higher eukaryotes, Sun1 localizes to NPCs and is required for de novo NPC assembly, which also requires NE fenestration (Doucet and Hetzer, 2010; Funakoshi et al., 2011; Talamas and Hetzer, 2011). Mechanistically, it is unclear how SUN proteins lead to NE remodeling. The luminal linkage of LINC complexes could drive INM and ONM approximation, and closing of the hairpin might drive membrane closure to form an NE fenestra directly or through recruitment of other factors (Fig. 5 C). In support of this model, our binding data suggest that both N and C termini of Mps2 and Mps3 interact before SPB insertion, but we have been unable to definitively determine if C-terminal binding occurs before N-terminal binding, as both mutant data and our model suggest.

At the SPB, our data call into question the common view that a Bbp1-containing complex encircles the SPB in wild-type cells to anchor it in the NE. Our results indicate that Mps3 is a fifth SPIN component and that it, rather than Bbp1, is a major component of the toroid. Why Bbp1 is restricted in most cells is not understood; its spatial regulation may be important to separate kinases such as Polo (Cdc5) from potential targets, as Cdc5 is recruited to the SPB by Bbp1 (Park et al., 2004).

## Materials and methods

### Yeast strains

Yeast strains are derivatives of W303 and are listed in Table S1. Standard conditions were used for yeast growth (Dunham et al., 2015). Deletion and tagging of genes was done using PCR-based methods in SLJ1070 (*Mata/Matα bar1/bar1 ade2-1/ADE2 trp1-1/TRP1 lys2Δ/LYS2 leu2-3,112/leu2-3,112 his3-11,15/his3-11,15 ura3-1/ura3-1*) and was verified by PCR (Longtine et al., 1998; Sheff and Thorn, 2004; Gardner and Jaspersen, 2014). PCR primers for gene deletion were designed as follows: F1 primer-60 bp upstream of gene-specific start codon followed by 5′-CGG​ATC​CCC​GGG​TTA​ATT​AA-3′; R1 primer-60 bp downstream of gene specific stop codon on the reverse strand followed by 5′-TCG​ATG​AAT​TCG​AGC​TCG​T-3′. PCR primers to tag genes at the C terminus were designed as follows: F5 primer-60 bp of gene-specific sequence immediately before the stop codon followed by 5′-GGT​GAC​GGT​GCT​GGT​TTA-3′; R3 primer-60 bp of gene specific sequence immediately after the stop codon on the reverse strand followed by 5′-TCG​ATG​AAT​TCG​AGC​TCG-3′. PCR primers to tag genes at the N terminus were designed as follows: F primer-60 bp of gene-specific sequence up to the start codon followed by 5′-GGT​CGA​CGG​ATC​CCC​GGG​TT-3′; R primer-60 bp of gene specific sequence immediately after the start codon on the reverse strand followed by 5′-AGA​ACC​ACC​ACC​AGA​ACC​AC-3′. Strains were made homozygous by tetrad dissection, which allowed us to verify that fluorescent protein fusions did not have obvious effects on cell growth when expressed as the sole copy in the cell (unpublished data).

SPB diameter scales with the ploidy of yeast: from 90 to 110 nm in haploids to 160 nm in diploids (Byers and Goetsch, 1975; Adams and Kilmartin, 2000). As the resolution limit for SIM is ∼100 nm, rings are difficult to detect in haploid cells. To increase SPB diameter and thus the diameter of the toroid to facilitate its detection, strains for SIM experiments were made into homozygous diploids by crosses. In some cases, diploids arose spontaneously, presumably because tagged combinations of SPB components or the deletion resulted in a subtle defect in some aspect of spindle formation. For other experiments, haploid strains were used unless we encountered spontaneous diploidization, then all strains for a given experiment were diploid. The ploidy of all strains was verified by flow cytometric analysis of DNA content at the time of strain construction and when strains were grown for imaging experiments.

Construction of strains containing *mps2-381* or *mps3Δ2-150*/*mps3-F592S* has been previously reported (Jaspersen et al., 2006; Bupp et al., 2007). Double mutants were created using standard genetic methods (Dunham et al., 2015). *mps3* mutants were created by oligonucleotide-directed mutagenesis of pSJ154 (pRS314-*MPS3*) and transformed into *mps3Δ::HIS3MX pURA3-MPS3* (SLJ1053).

The N-terminal Mps2 reporter, pSJ2165 (pRS315-*NOP1pr-GFP_11_-mCh-MPS2*), was created by cloning a PCR fragment containing the *MPS2* ORF into the NheI and SalI sites of the nuclear reporter pSJ1321 (pRS315-*NOP1pr-GFP_11_-mCh-PUS1*; Smoyer et al., 2016). To construct the C-terminal Mps2 reporter, pSJ2449 (pRS315-*NOP1pr-mCh-MPS2-GFP_11_*), a C-terminal fragment containing *MPS2-GFP_11_*, was made by gene synthesis and inserted into pSJ2165 at StuI and SalI sites; the N-terminal GFP_11_ was removed by digestion with ApaI and XhoI followed by religation. Construction of the ONM/ER reporter pSJ1568 (pRS315-*NOP1pr-GFP_11_-mCherry*-*SCS2TM*) and the luminal reporter pSJ1602 (pRS316-*NOP1pr-mCherry-SCS2TM*-*GFP_11_*) has been described (Smoyer et al., 2016). A diploid strain containing each reporter was created by transformation, and proteins were fused to the coding sequence for GFP_1–10_ by PCR using N- and C-terminal tagging constructs as previously described (Smoyer et al., 2016). Haploid cells containing both halves of split-GFP were generated by sporulation followed by tetrad dissection.

### Yeast two-hybrid interactions

Yeast two-hybrid interactions were tested using low-copy centromeric plasmids expressing fusion proteins from the *ADH1* promoter (Uetz et al., 2000). Strains SLJ1644 (wild-type), SLJ12623 (*pom152Δ*), and SLJ12624 (*pom152Δ mps2Δ*) were cotransformed with binding- and activation-domain fused plasmids, pOBD-Bbp1 (pSJ403), pOAD-Ndc1 (pSJ383), and pOAD-Spc29 (pSJ1828), respectively. Transformants were selected on SD-Leu-Trp plates. To test for interactions, two OD_600_ of cells from each strain were serially diluted 10-fold, and ∼10 µl of each dilution was spotted on SD-Leu-Trp or SD-Leu-Trp-His plates containing 25 mM 3-amino-triazole (3AT; Sigma-Aldrich; A8056). Plates were incubated at 30°C for 4 d. Growth on SD-Leu-Trp-His + 3AT indicates an interaction.

### SIM imaging

Cells were grown to an OD_600_ of 0.5–0.8 in freshly prepared imaging medium (6.7 g yeast-nitrogen base with ammonium sulfate without amino acids, 5 g casamino acids, 16.6 mg uracil, and 950 ml ddH_2_O; after autoclaving, 4 ml of 4 mg/ml adenine, 2 ml of 4 mg/ml tryptophan, and 50 ml 40% [wt/vol] sterile glucose were added). Cells were fixed for 15 min in 4% PFA (Ted Pella; 18505) in 100 mM sucrose, then washed two times in PBS, pH 7.4. An aliquot of cells was resuspended in Dako mounting medium (Agilent Technologies; S3023), placed on a cleaned glass slide, covered with a number 1.5 coverslip, and then allowed to cure overnight at room temperature.

SIM images were acquired with an Applied Precision OMX BLAZE (GE Healthcare) equipped with an Olympus 60× 1.42-NA Plan Apo oil objective. Images were collected in sequential mode with two or three PCO Edge sCMOS cameras for each acquisition channel. Color alignment from different cameras in the radial plane was performed using the color alignment slide from GE Healthcare. In the axial direction, color alignment was performed using 100-nm TetraSpeck beads (Thermo Fisher Scientific; F8803). Reconstruction was accomplished with softWoRx v6.52 software (GE Healthcare) according to the manufacturer’s recommendations with a Wiener filter of 0.001. In most cases, images are mT2/YFP with 514-nm excitation for YFP and then 445-nm excitation for mT2. In some cases, we modified the protocol for mT2/YFP/mCherry acquisition with the mCherry acquired first and excited with the 568-nm laser. The dichroic in every case was 445/514/561, with emission filters at 460–485, 530–552, and 590–628 nm for mT2, YFP, and mCherry, respectively.

Rings generally appeared incomplete in a single z-plane (Fig. S1 B), possibly due to random orientation of the SPB (and thus the toroid) in 3D, nonrandom incomplete distribution of SPIN components at the toroid, or localized fluorophore quenching/activation or other photon effects. To account for these issues, Ndc1-YFP was used as a fiducial marker to locate toroids, since Ndc1 is essential for SPB insertion and has previously been observed at toroids using SIM (Chial et al., 1998; Burns et al., 2015; Rüthnick et al., 2017). If we detected a Ndc1-YFP ring/ring-like structure and the ring was not in the top or bottom slice of the z-stack, we examined the distribution of other SPIN components/Mps3 and used images for SPA-SIM analysis; if a Ndc1-YFP ring was not observed, we did not score that cell. For image preparation, the SIM reconstructed images were scaled 4 × 4, and a maximum projection in z over the relevant slices was done.

### SPA-SIM

All single-particle averaging was performed using custom macros and plugins for the open source program ImageJ (National Institutes of Health). Plugins and source code are available for download at http://research.stowers.org/imagejplugins. Toroid alignment is fundamentally different from the multispot alignment we previously described (Burns et al., 2015; Bestul et al., 2017) and therefore requires a different fitting strategy. For toroid alignment, we exclusively used Ndc1-YFP as our fiducial marker except in split-GFP experiments, where we used mCherry signal associated with Mps2. Given the often incomplete appearance of toroids from 3D SIM microscopy (Fig. S1 B), we need a method to globally fit multiple parts of the ring simultaneously to a model that accounts for 3D positioning of the ring as well as its rotation around the z axis of the microscope (φ) and its tilt with respect to that axis (θ).

We begin with the mathematical description of the ring itself. The following equation describes the travel in Cartesian space (x, y, z) around a ring of radius *r*, which is tilted from the z axis by angle θ and then rotated about the z axis by angle ɸ:

x(ρ)=xc−r⋅cos(ρ)⋅sin(φ)+r⋅sin(ρ)⋅cos(θ)⋅cos(φ),

y(ρ)=yc+r⋅cos(ρ)⋅cos(φ)+r⋅sin(ρ)⋅cos(θ)⋅sin(φ),

z(ρ)=zc−r⋅sin(ρ)⋅sin(θ). (1)

Here, *xc*, *yc*, and *zc* are the center of the ring in 3D, and ρ is the angle that has been traveled about the ring.

Experimentally, a robust way to determine the positioning and orientation of a ring is to examine its xz cross sections from its approximate center at multiple angles (in our case, we used 0°, 45°, 90°, and 135°; see Fig. S1 A). Because of the asymmetric resolution of the microscope, each point where the ring crosses each xz cross section is an asymmetric Gaussian function whose lateral dimension is the approximate lateral resolution of the microscope and whose vertical dimension is the approximate axial (z) resolution of the microscope. To improve the statistical accuracy of our cross sections, we average over a 2-pixel-wide region for the xz cross sections.

Our task is now to fit a set of eight cross-sectional spots using the tilted ring model described above. If we treat the initial guess of the center position of the ring as the origin, we must simply find the ρ values (and therefore 3D coordinates) at which the ring crosses the xz plane, the yz plane, and the diagonal planes at 45° and 135°. This is done by solving Eq. 1 for *x* = 0 (xz plane crossings), *y* = 0 (yz plane crossings), *x* = *y* (45° crossings), and *x* = −*y* (135° crossings). The solutions were found with the aid of the Mathematica program (Wolfram) as follows:

axz=sin(φ)cos(θ)⋅cos(φ),

cxz=xcr⋅cos(θ)⋅cos(φ),

ρxz=tan−1(−cxz±axz⋅1+axz2−cxz2axz⋅cxz±1+axz2−cxz2), (2)

ayz=cos(φ)cos(θ)⋅sin(φ),

cyz=ycr⋅cos(θ)⋅sin(φ),

ρyz=tan−1(−cyz±ayz⋅1+ayz2−cyz2ayz⋅cyz∓1+ayz2−cyz2), (3)

a45=sin(φ)+cos(φ)cos(θ)⋅sin(φ)−cos(θ)⋅cos(φ),

c45=xc−ycr⋅[cos(θ)⋅sin(φ)−cos(θ)⋅cos(φ)],

ρ45=tan−1(c45±a45⋅1+a452−c452a45⋅c45∓1+a452−c452), (4)

a135=sin(φ)−cos(φ)−cos(θ)⋅sin(φ)−cos(θ)⋅cos(φ),

c135=xc+ycr⋅[−cos(θ)⋅sin(φ)−cos(θ)⋅cos(φ)],

ρ135=tan−1(c135±a135⋅1+a1352−c1352a135⋅c135∓1+a1352−c1352). (5)

Care was taken to ensure that ρ values were between 0 and 2π and that the crossing points were not swapped. The final fit was a nonlinear least squares global fit (Bevington and Robinson, 2003) to eight asymmetric 2D Gaussians. The standard deviations of these Gaussians in x and z were linked together, and the amplitudes on either side of each cross section were constrained to be no more than a factor of 2 different from one another. Four points were manually placed on the image at the approximate locations of the 0°, 90°, 180°, and 270° crossing points of the ring. The radius was initialized from the average of the two distances derived from these points, and the center in x and y was initialed at the center of these four points. The center in z was initialized at the maximum-intensity position of the average of all of the z intensity profiles at these four points. The center of the toroid was constrained to be within 20% of the guess radius from the initialized center, and the radius itself was constrained to be within a factor of 2 of its original initialized value. The z position was constrained to be within one z slice from its initialized position. The tilt angle (θ) was constrained to values <45°. At low tilt angle values (for essentially flat rings), the rotation angle (ɸ) is poorly defined. As a result, we fixed the rotation angle (ɸ) at 10° increments and repeated the fit for every possible value of ɸ to ensure the best fit.

Quality of fit was assessed by visual inspection of the fitted cross-sectional images in comparison to the final fit in simulated cross-sectional images. The original 3D image was then transformed so that the fitted toroid was flat at the center of the final transformed image. In some cases, after determining that gaps in rings did not align, the final images were randomly rotated about their centers (in the xy plane) to avoid accidental nonhomogeneous regions in the aligned image. The final SPA-SIM image was formed by averaging the realigned images. In rare cases, one image was much brighter than the others in a series; when this occurred, the bright image was normalized by its ratio to the other image intensities to avoid that image dominating the averaged image.

There is reason to believe that the distribution of the secondary (not fiducial) channel is oriented at a specific direction from the center of the toroid in some cases (e.g., Mps3, which is localized on the half-bridge; Jaspersen et al., 2002). For these, the angle of each individual toroid’s rotation was determined by manual drawing of a line between the ring center and the secondary distribution center. Images were then rotated so that the asymmetry is pointing either upwards or sideways before averaging. These cases are specifically pointed out in the text.

Radial profiles were generated using custom software interpolating pixel values at 1-pixel arc lengths and averaging around ever-expanding circles from the center of each aligned averaged image. Diameters were determined by fitting intensity profiles drawn through the vertical and horizontal centers of the averaged ring images to two Gaussian functions. Errors were determined by Monte Carlo analysis as described in Burns et al. (2015). In cases where toroids appear approximately symmetric, the reported diameters were the average of vertical and horizontal values with propagated errors. In asymmetric cases (e.g., Mps3), we independently report the vertical and horizontal diameters. Significance testing for ring diameter differences was performed with a two-tailed *t* test on the Monte Carlo–derived diameter distributions. Data distribution was assumed to be normal, but this was not formally tested.

### FRET

Cells were grown and fixed identically to SIM samples. An aliquot of fixed cells was resuspended in ProLong Diamond Antifade mounting media (Thermo Fisher Scientific; P36961), placed on a cleaned glass slide, covered with a number 1.5 coverslip, and then allowed to cure overnight at room temperature. Images were acquired on a Nikon Eclipse TI equipped with a Yokogawa CSU W1 spinning disk head and Andor EMCCD using a Nikon Apo TIRF 100× 1.49-NA oil objective. mT2 was imaged using a 445-nm laser and 480/30 emission filter with a maximum power of 1.2 mW measured at the sample. YFP was imaged using a 514-nm laser and ET535/30m emission filter with a maximum power of 2.5 mW measured at the sample. For each sample, 16 points were manually or automatically selected depending on cell density. Afterwards, an automation script moved to positions, found focus using Nikon PFS, imaged mT2/YFP, bleached at 514 nm for 1 min, and reimaged. Image processing was performed in ImageJ using custom macros and plugins (https://github.com/jouyun/). In brief, a small blurring was performed, followed by a background subtraction and registration; puncta were identified using a local maximum finder and adaptive region grow; these were quantified in the mT2 channel before and after the bleach. Average FRET values with donor only and an acceptor and donor pair were used to determine the relative FRET efficiency. Errors were propagated to determine error bars for the relative FRET efficiency, and statistical significance was determined using a two-sided Student’s *t* test. Data distribution was assumed to be normal, but this was not formally tested.

### BiFC assay

Cells were grown overnight at 23°C in SD-Leu to mid-log phase. Samples were immobilized between a slide and a number 1.5 coverslip before imaging with a 40×, 1.2-NA, Plan-apochromatic objective on a Zeiss LSM780 equipped with a Quasar multichannel spectral Gallium arsenide phosphide (GaAsP) detector and Zeiss Zen Black software. Imaging was conducted in multitrack mode, with the pinhole set to 1 airy unit, pixel dwell time 12.5 µs, and pixel xy scaling 0.104 µm. Green fluorescence was collected at wavelengths of 491–562 nm while exciting using a 488-nm argon laser line at 8% power. Red fluorescence was collected at wavelengths of 571–695 nm while exciting using a 561-nm argon laser line at 0.6% power. A total of five z-slices were collected per sample at 0.540 µm spacing. Images were scaled 4 × 4, and a maximum projection in z over two to five slices was done with ImageJ.

For split-GFP with SIM, samples were grown, fixed, and mounted as described above, then mCherry/GFP was imaged with 568-nm excitation for mCherry and then 488-nm excitation for GFP using an Applied Precision OMX BLAZE (GE Healthcare) equipped with a 60× 1.42-NA Plan Apo oil objective. The dichroic in every case was 568/448 with emission filters at 590–628 nm and 504–552 nm for mCherry and GFP, respectively. Note that the pixelated appearance of mCherry-GFP_11_-Pus1 in SIM images is due to the SIM reconstruction, as it is not observed in confocal images (Smoyer et al., 2016).

Three-color data, including mCherry, split-GFP, and mT2 as shown in Fig. 5, were acquired on a Leica SP8 microscope with 100×, 1.4-NA oil objective. mCherry, split-GFP, and mT2 were excited using laser lines at 561, 496, and 445 nm. A nonoptimal 496-nm laser line was used for split-GFP to minimize bleed-through from mT2 (based on published spectra in https://www.FPbase.org, the mT2 absorption efficiency at 496 nm is ∼1/30 of the value it is at 488 nm, effectively eliminating cross-talk). All emission photons were collected by an internal Leica HyD hybrid detector with spectral windows of 463–495 nm for mTurquiose2, 508–550 nm for split-GFP, and 570–635 nm for mCherry. All images were acquired under the HyVolution setup in the Leica software, which uses a pinhole setting at 0.57 Airy units along with deconvolution to improve lateral resolution up to 140 nm. Raw images were deconvolved with Huygens Software via the Leica interface with the default setting. For postprocessing, images were smoothed in ImageJ with a Gaussian blur of radius 1 pixel and scaled 2 × 2 with bilinear interpolation.

### Transmission EM

Cells were high-pressure frozen, freeze substituted, sectioned, and stained as previously described to examine the SPB by EM (Giddings et al., 2001; Jaspersen et al., 2002). Serial thin sections were viewed on a Philips CM10 electron microscope, and images were captured with a Gatan digital camera and viewed with Digital Micrograph software.

### SPB insertion assay

For characterization of SPB duplication using the red/green foci assay, images were acquired with a 100× 1.4-NA oil objective on an inverted Zeiss 200m equipped with a Yokagawa CSU-10 spinning disc. 488-nm excitation and 568-nm excitation were used for GFP and mCherry, respectively, and emission was collected through BP 500–550-nm and BP 590–650-nm filters, respectively, onto a Hamamatsu EMCCD (C9000-13). For each channel, a z-stack was acquired using 0.6- or 0.7-µm spacing. 13 total slices were acquired, and a maximum-projection image was created for analysis of foci using ImageJ.

### Original data

Original data underlying this manuscript can be downloaded from the Stowers Original Data Repository at http://www.stowers.org/research/publications/LIBPB-1349.

### Online supplemental material

Fig. S1 shows 3D single-particle averaging of toroidally distributed proteins. Fig. S2 shows Mps3, mutants, and interaction with SPIN components. Fig. S3 shows that loss of Mps2 specifically disrupts Mps3 at the toroid. Table S1 lists yeast strains.

## Supplementary Material

Supplemental Materials (PDF)

Table S1 (Excel)
