# Supplementary material for: Yeast centrosome components form a noncanonical LINC complex at the nuclear envelope insertion site
Source: J Cell Biol. 2019 Mar 12;218(5):1478–90. doi: 10.1083/jcb.201809045 (PMC6504903; doi:10.1083/jcb.201809045)
Supplement: Supplemental Materials (PDF) [file JCB_201809045_sm.pdf]

Supplemental material

Chen et al., https://doi.org/10.1083/jcb.201809045

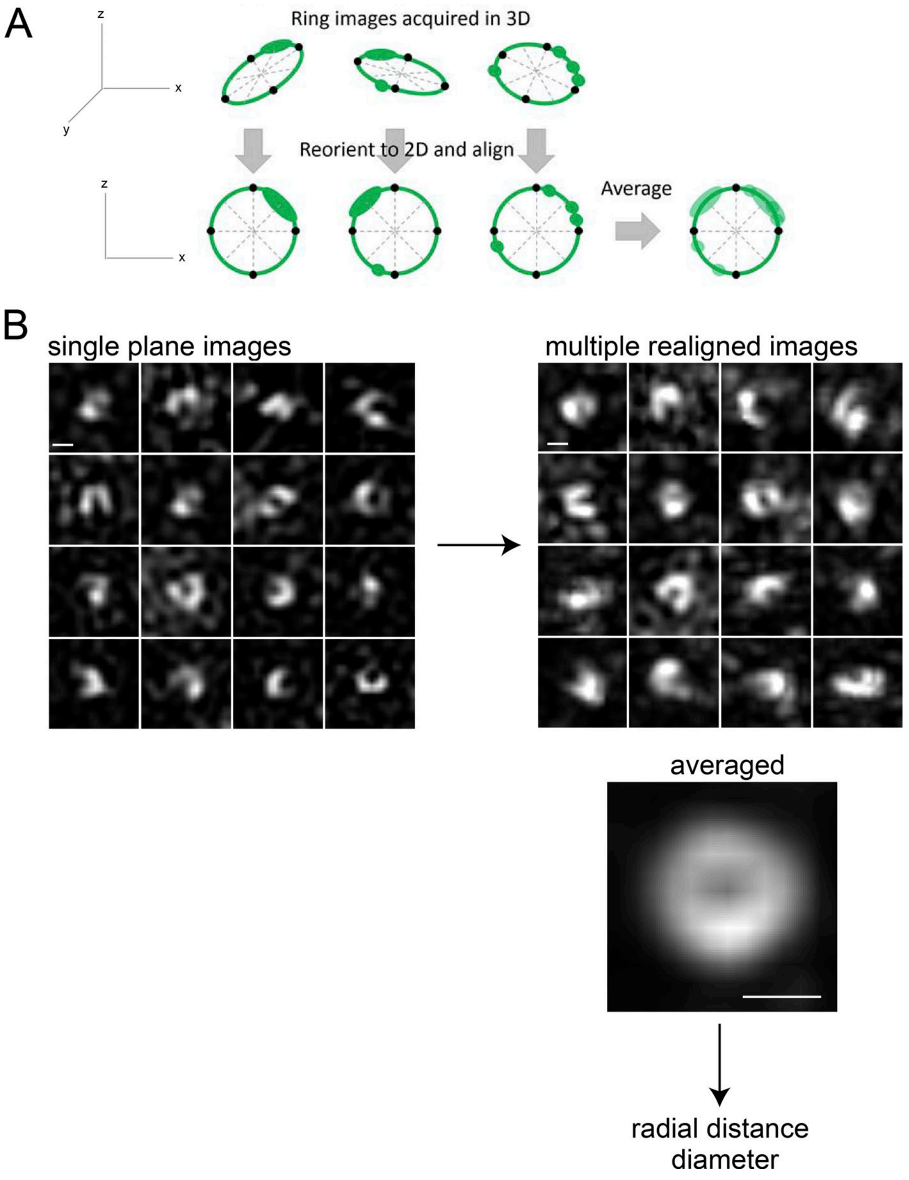

Figure S1. **3D single-particle averaging of toroidally distributed proteins. (A)** SPBs, and therefore toroids, are randomly oriented in 3D space. To compare the distribution of proteins, we computationally reoriented each ring so that the toroid was in cross section in the xz plane. This allowed us to create averaged images that incorporate the likelihood of finding a pixel at each position based on all images. **(B)** First, a montage of single plane SIM images of Ndc1-YFP is shown. Note how the ring lies in a single plane in some, while in others, one or two dots are seen. Next, images were realigned, making rings in individual images more apparent, and then averaged to create a projection. From this, radial intensity profiles from the center of the SPB outward can be determined and used to estimate diameter. Except in the case of Mps3, which shows clear asymmetry, we assumed that the ring was homogeneous for averaging purposes despite the appearance of heterogeneity in individual images. Heterogeneity is not atypical for superresolved ring structures approaching this scale (e.g., Mennella et al., 2012; Szymborska et al., 2013; Gartenmann et al., 2017), possibly due to photon statistics, reconstruction noise (including "hammerstroke" noise; Demmerle et al., 2017), stochastic bleaching, and variation in fluorophore emission. We do not believe the heterogeneity is the result of realignment, as it is present in unaligned rings, nor is it likely the result of actual biological heterogeneity, as illustrated by the random relationship between Ndc1 heterogeneity and the orientation of Mps3 (shown in Fig. 2, A, H, J, and L). Other than for Mps3, we represented averaged pore images throughout the article based on random orientation of the ring, since it is the simplest choice based on available data. Bars, 100 nm.

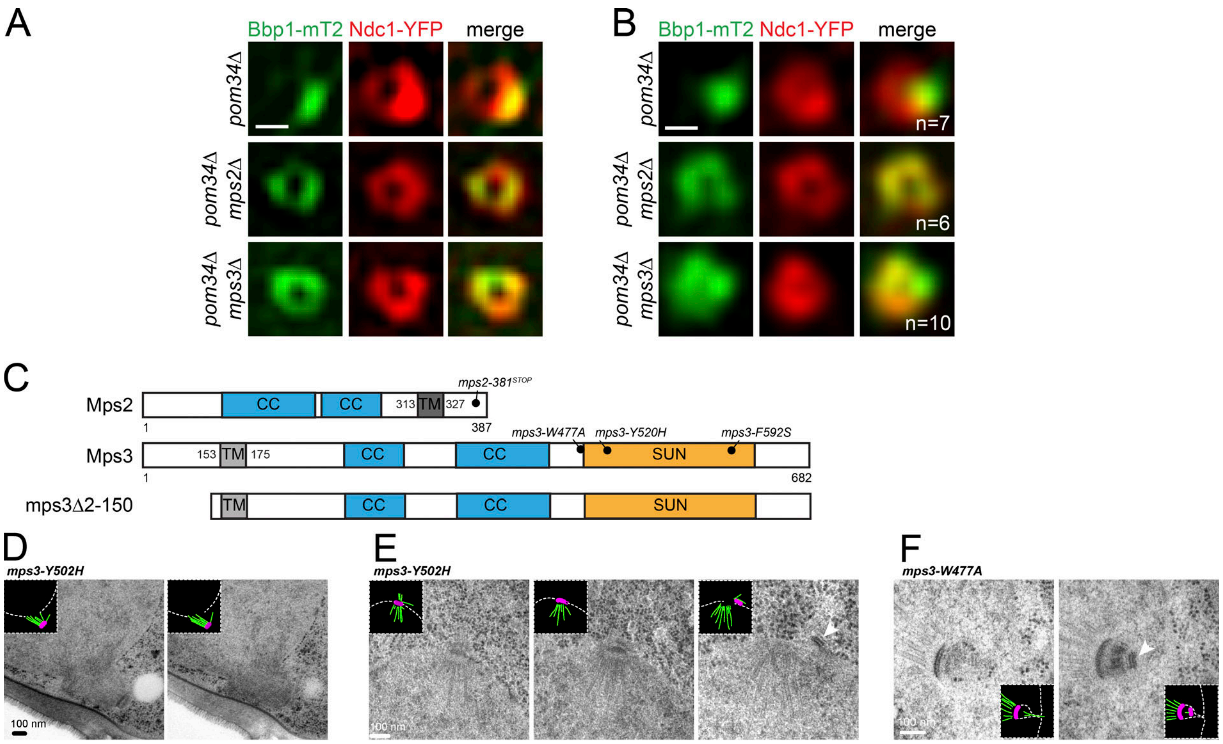

G

	acceptor (YFP)					
**donor (mT2)**	Ndc1-C	Mps2-C	Nbp1-C	Bbp1-C	N-Mps3	Mps3-C
Ndc1-C		1.5±0.9 (n=299)	-2.9±1.3 (n=233)	ND	-5.7±1.5 (n=213)	ND
N-Mps2	ND*		**14.9±2.5 (n=80)**	**8.0±1.9 (n=118)**	ND*	ND
Nbp1-C	**9.0±1.7 (n=146)**	ND		-2.4±0.7 (n=587)	**5.7±0.7 (n=795)**	ND
Bbp1-C	**12.5±1.3 (n=197)**	ND	**9.6±1.1 (n=385)**		2.6±1.3 (n=224)	ND
Mps2-C	ND	ND	ND	ND	ND	**39.9±2.1 (n=271)**

Figure S2. **Mps3, mutants, and interaction with SPIN components. (A and B)** Localization of Ndc1-YFP (red) and Bbp1-mT2 (green) in individual SIM images (A) or averaged images (B) from *pom34Δ* (SLJ12300), *pom34Δ mps2Δ* (SLJ10936), and *pom34Δ mps3Δ* (SLJ11913). Number of SPBs included in average is shown. **(C)** Schematic of Mps2 and Mps3 showing the position of coiled-coil and transmembrane domains. The Mps3 SUN domain is also shown. Loss-of-function mutants in *MPS2* and *MPS3* (Jaspersen et al., 2006) and a schematic of *mps3Δ2-150* are shown. **(D–F)** Serial section EM images and a schematic showing SPBs and the NE from mutants in the Mps3 SUN domain. In D, the SPB is not anchored in the NE in *mps3-Y502H*. In E and F, the SPB from *mps3-Y502H* (E) or *mps3-W477A* (F) has duplicated, but the new SPB cannot insert into the NE (arrow, right panels). Bars, 100 nm. **(G)** Acceptor photobleaching FRET between SPIN components and with Mps3. Average FRET efficiency is listed along with SEM; the number of cells analyzed is listed. Some FRET pairs were not examined because of protein topology (ND) or incompatibility between tagged proteins (ND*).

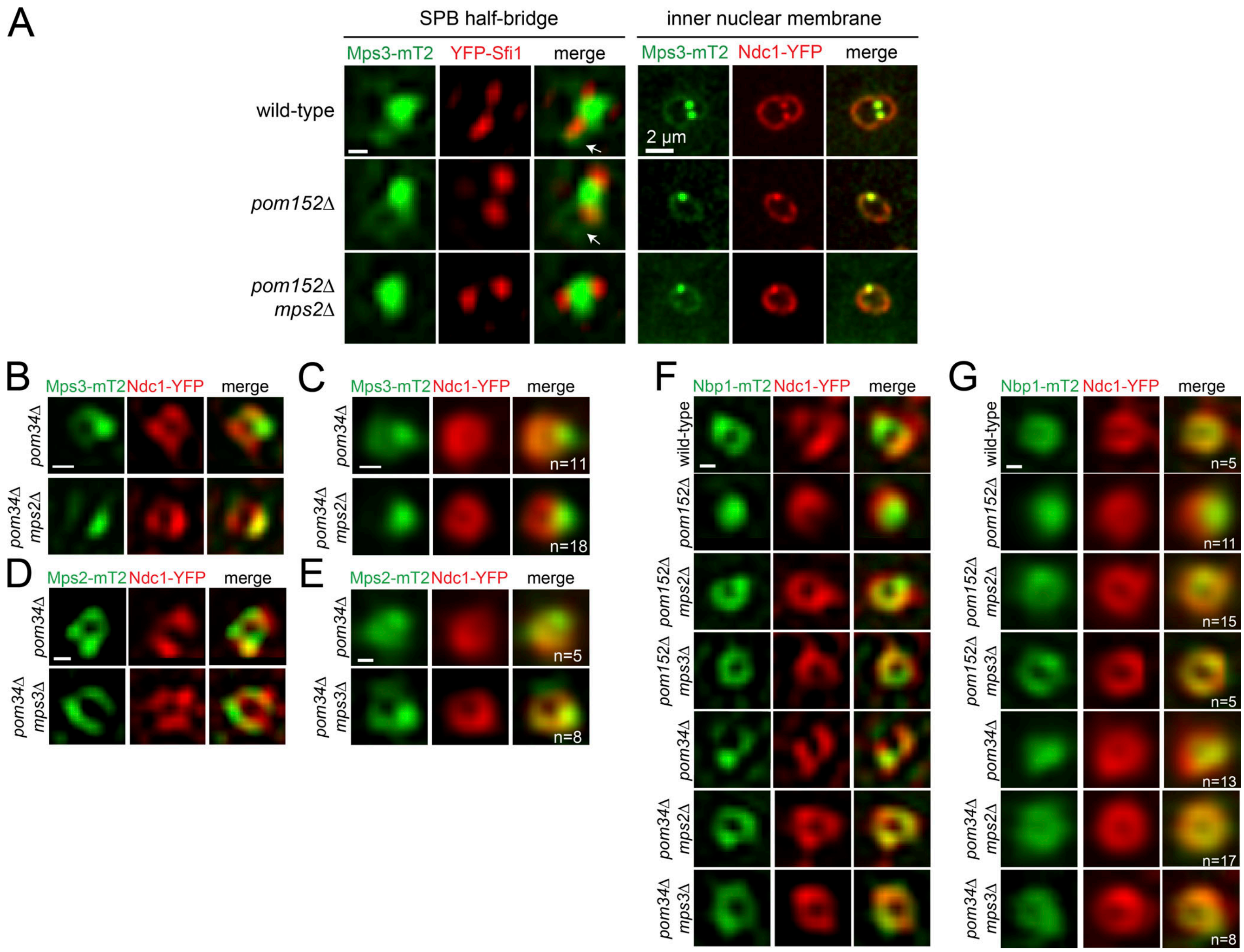

Figure S3. **Loss of Mps2 specifically disrupts Mps3 at the toroid. (A)** SIM showing localization of YFP-Sfi1 (red) and Mps3-mT2 (green) in G1 cells selected from an asynchronously growing population of wild-type (SLJ12060), *pom152Δ* (SLJ12100), *or pom152Δ mps2Δ* (SLJ12101) cells. Arrows point to Mps3-mT2 that can be seen around one YFP-Sfi1 focus, which is likely to be the mother SPB, in wild-type (from Fig. 2 D) and *pom152Δ* but not *pom152Δ mps2Δ* cells. Localization of Ndc1-YFP (red) and Mps3-mT2 (green) in G1 cells selected from an asynchronously growing population of wild-type (SLJ10636), *pom152Δ* (SLJ11071), and *pom152Δ mps2Δ* (SLJ10535). Bar, 100 nm (left); 2 µm (right). **(B and C)** Individual SIM (B) and averaged (C) images showing localization of Ndc1-YFP (red) and the distribution of Mps3-mT2 (green) in *pom34Δ* (SLJ11165) and *pom34Δ mps2Δ* (SLJ10899). **(D and E)** Individual SIM (D) and averaged (E) images showing localization of Ndc1-YFP (red) and the distribution of Mps2-mT2 (green) in *pom34Δ* (SLJ12303) and *pom34Δ mps3Δ* (SLJ11968). **(F and G)** Localization of Ndc1-YFP (red) and Nbp1-mT2 (green) in individual SIM images (F) and averaged images (G) from wild-type (SLJ10898), *pom152Δ* (SLJ12301), *pom152Δ mps2Δ* (SLJ10863), *pom152Δ mps3Δ* (SLJ10864), *pom34Δ* (SLJ11970), *pom34Δ mps2Δ* (SLJ10900), and *pom34Δ mps3Δ* (SLJ11969) strains. Number of SPBs included in average is shown. Bars, 100 nm.

Provided online is one table in Excel. Table S1 lists yeast strains.

References

Demmerle, J., C. Innocent, A.J. North, G. Ball, M. Müller, E. Miron, A. Matsuda, I.M. Dobbie, Y. Markaki, and L. Schermelleh. 2017. Strategic and practical guidelines for successful structured illumination microscopy. *Nat. Protoc.* 12:988–1010. https://doi.org/10.1038/nprot.2017.019

Gartenmann, L., A. Wainman, M. Qurashi, R. Kaufmann, S. Schubert, J.W. Raff, and I.M. Dobbie. 2017. A combined 3D-SIM/SMLM approach allows centriole proteins to be localized with a precision of ~4-5 nm. *Curr. Biol.* 27:R1054–R1055. https://doi.org/10.1016/j.cub.2017.08.009

Jaspersen, S.L., A.E. Martin, G. Glazko, T.H. Giddings, G. Morgan, A. Mushegian, and M. Winey. 2006. The Sad1-UNC-84 homology domain in Mps3 interacts with Mps2 to connect the spindle pole body with the nuclear envelope. *J. Cell Biol.* 174:665–675. https://doi.org/10.1083/jcb.200601062

Mennella, V., B. Keszthelyi, K.L. McDonald, B. Chhun, F. Kan, G.C. Rogers, B. Huang, and D.A. Agard. 2012. Subdiffraction-resolution fluorescence microscopy reveals a domain of the centrosome critical for pericentriolar material organization. *Nat. Cell Biol.* 14:1159–1168. https://doi.org/10.1038/ncb2597

Szymborska, A., A. de Marco, N. Daigle, V.C. Cordes, J.A. Briggs, and J. Ellenberg. 2013. Nuclear pore scaffold structure analyzed by super-resolution microscopy and particle averaging. *Science.* 341:655–658. https://doi.org/10.1126/science.1240672
